# Risk factors and clinical relevance of positive urine cultures in cats with subcutaneous ureteral bypass

**DOI:** 10.1186/s12917-021-02898-7

**Published:** 2021-05-27

**Authors:** Julie Deprey, Arnaud Baldinger, Véronique Livet, Margaux Blondel, Mathieu Taroni, Cynthia Lefebvre, Isabelle Goy-Thollot, Pierre Moissonnier, Éric Viguier, Céline Pouzot-Nevoret, Claude Carozzo, Thibaut Cachon

**Affiliations:** 1 Department of Small Animal Surgery, Vetagro Sup, Campus Vétérinaire de Lyon, Marcy l’Etoile, France; 2 Research Unit ICE, UPSP 2011.03.101, Université de Lyon, Veterinary Campus of VetAgro Sup, Marcy l’Etoile, France; 3 Intensive Care Unit (SIAMU), Université de Lyon, Veterinary Campus of VetAgro Sup, Marcy l’Etoile, France; 4 Université de Lyon, Veterinary Campus of VetAgro Sup, APCSe, F-69280 Marcy l’Etoile, France

**Keywords:** Feline, Ureteral obstruction, Urinary tract infection, Subcutaneous ureteral bypass

## Abstract

**Background:**

The objective of the study was to report the incidence and risk factors associated with positive urine bacterial cultures as well as long-term outcome in cats with subcutaneous ureteral bypass (SUB) devices.

**Results:**

Medical records of cats that underwent SUB device placement were retrospectively reviewed. Signalment of the cat, laterality of the ureteral obstruction, surgery, anesthesia and hospitalization duration, bacterial culture results and follow-up data were retrieved.

Thirty-two cats met the inclusion criteria. Four cats (12.5%) had a positive intraoperative culture, with two of them being treated successfully. Ten cats out of 28 (35.7%) were documented with a positive urine culture during follow-up period, with a median time between discharge and identification of the first positive urine culture of 159 days (range 8–703 days). Bacteriuria resolved in 60% of cats (6/10). *Escherichia coli* was the most common organism, isolated in 4 out of 10 postoperative urine cultures. Overall, subclinical bacteriura was documented for 6 of 32 (18.8%) cats and 5 of 32 (15.6%) cats displayed clinicals signs suggestive of persistent UTI. One cat had subclinical bacteriuria. Three cats died during the follow-up period.

There was a significant difference between negative and positive urine bacterial culture groups in median hospitalization duration (5 days versus 6 days, *P* = 0.022) and in median body condition score (5/9 versus 4/9, *P* = 0.03). Cats with a longer hospital stay and with a lower body condition score were more likely to have a positive urine culture during follow-up period.

**Conclusions:**

SUB device placement surgery is associated with complications such as chronic bacteriuria. Bacteriuria in our study resolved with appropriate antibiotic treatment in more than half of cats. Risk factors identified for positive urine culture were a longer hospitalization duration and a decreased body condition score.

## Background

Subcutaneous ureteral bypass (SUB) placement is an increasingly common procedure for renal decompression in cats with benign ureteral obstruction [1–6].

According to the study of Berent et al. including 134 cats, SUB placement is associated with short term complications including device occlusion with blood clots (8.1%), kinking of the tubing device (4.6%) and device leakage (3.5%), and long-term complications, the most common being catheter mineralization (24.2%) [1]. Development of bacteriuria following device placement is documented by positive urine culture from 21 to 30.8% of cats [1, 2, 6, 7]. Infections were successfully cleared with an appropriate antibiotic treatment in 50 to 78% of cats. 13 to 50% of cats had persistant bacteriuria, diagnosed as lower urinary tract signs and a persisting positive urine culture despite appropriate treatment based on microbial culture [1, 2, 6]. Cats with a positive urine culture at the time of SUB device placement were significantly more likely to have bacteriuria after the surgery and to have persistant bacteriuria in one study [1].

The purpose of the study was to report the incidence and risk factors associated with positive intraoperative and postoperative urine cultures and to determine the long-term outcome in cats with subcutaneous ureteral bypass. We hypothesized that positive intraoperative urine cultures would be related to the development of signs consistent with UTI during the follow-up period. We also hypothesized that duration of surgery and anesthesia would be associated with positive urine bacterial cultures.

## Methods

### Case selection

Medical records (January 2014 – February 2019) of cats treated for ureterolithiasis with ureteral obstruction with a SUB device at VetAgro-Sup were reviewed. A urine culture performed intraoperatively from a pyelocentesis and 1 and 3 months postoperatively through the SUB port were also needed for inclusion. If a positive urine bacterial culture was documented, another urine bacterial culture was needed after appropriate antimicrobial treatment for inclusion. A culture-negative group and a culture-positive group were created to identify risks factors. The minimal follow-up period to be included was 6 months.

### Data

Information retrieved included signalment (breed, sex and age), weight, body condition score (BCS), initial and follow-up biochemical values, affected kidneys, operative and anesthetic times, intraoperative urine culture results, bacterial species isolated, duration, administration of antibiotic treatment including prophylactic parenteral administration of antimicrobial in the immediate proximity of surgery, or antimicrobial gave postoperatively, antimicrobial type used, preoperative and postoperative antimicrobial route of administration, time between surgery and discharge. At the end of the follow-up period, the number of animals with persistent ITU and cause of death were noted.

### Surgical procedure

As previously described [2], the SUB device was placed according to the surgical guide provided by Norfolk Vet Products [8] on the affected side. However, the kidney catheter was placed without imaging control. For each cat, urine was sampled from the renal pelvis for bacteriological culture. If the surgery was bilateral, urine samples were pooled.

### Postoperative management

Postoperative plain film radiographs were performed to ensure that no kinking of the device tubing was present. All patients received prophylactic antibiotic treatment (amoxicillin clavulanic acid 15 mg/kg PO q12h) for 7 days until reception of bacterial culture results. The antibiotic treatment was discontinued if the bacterial culture was negative. The antibiotic treatment was otherwise pursued in accordance with the sensitivity results for a total of 3 weeks.

### Follow-up

Following hospital discharge, the SUB device was flushed under ultrasound guidance 1 month and 3 months after surgery, and then every 3 to 6 months thereafter. As previously described [1], 0.5 mL of saline was flushed into the device, and the renal pelvis and the bladder were monitored for bubbles.

A serum biochemical analysis and urinalysis were performed at each follow-up. Culture of urine obtained from the SUB port was performed 1 and 3 months after surgery in all cats. In addition, during the follow-up period, if a cat was reported to have lower urinary signs (pollakiuria, dysuria, stranguria or hematuria), urine culture from the SUB port was performed. As for the post-operative period, an antibiotic treatment was initiated if the bacterial culture was positive.

### Statistical analysis

The Mann-Witney test was used to compare urine culture-positive and urine culture-negative groups for nonparametric continuous variables (age, BCS, surgery and anesthetic time, hospitalization duration, progression of the chronic kidney disease based on the International Renal Interest Society (IRIS) staging). The χ2 test or Fisher’s exact test was used to compare urine culture-positive and urine culture-negative groups for categorical variables (sex, SUB device type, presence or absence of bacteriuria intraoperatively, stage of acute kidney injury based on the International Renal Interest Society staging). A Kruskall-Wallis test was used to determine if hospitalization duration was associated with preoperative acute injury stage or SUB device type.

For all comparisons, a *P*-value was considered significant if <.05. All analyses were performed using commercial software (R - version 3.5.2).

Cats with positive intraoperative urine culture (*n* = 4) were not included to assess possible associations between positive urine bacterial culture and the above criteria.

## Results

Altogether, 48 cats underwent SUB device placement because of obstructive ureterolithiasis. Among them, 32 cats met the inclusion criteria. Sixteen cats were excluded from the study because the cat died before discharge (*n* = 3), data from follow-up visit to the referring veterinarian were missing (*n* = 7) or the cat was lost in follow up (*n* = 6). Twenty-one of 32 cats (66%) were domestic shorthair cats, other breeds were Birmans (*n* = 6), Persians (*n* = 2), Ragdoll (*n* = 2) and Chartreux (*n* = 1). Of the 32 cats, 18 (56%) were castrated males, 14 (44%) were spayed females. Median age, median body weight and median body condition score at the time of SUB device placement was 5.5 years (range 1.67–13.1 years), 3.9 kg (range 2.15–5.7 kg) and 5/9 (range 2/9–7/9) respectively. The median BUN concentration at admission was 117 mg/dL (range 28.6–327.6 mg/dL; reference range, 12 to 31 mg/dL) and median serum creatinine was 8.6 mg/dL (range 2.6–25.8 mg/dL; reference range, 0.6 to 2.1 mg/dL). Prior to discharge, median BUN concentration was 34.86 mg/dL (range 19.3–89.6 mg/dL) and median serum creatinine was 2.3 mg/dL (range 1.3–5.2 mg/dL). Eighteen of 32 cats (56%) had unilateral ureteral obstruction. Fourteen of 32 cats (44%) had bilateral ureteral obstruction. Of the 14 cats with bilateral SUB, 10 cats had a bilateral SUB device with a 3-way port and a single cystotomy tube. Overall median surgical procedure time was 102 min (range 50–150 min). The median duration of anesthesia was 160 min (range 110–215 min). Antibiotics were not administered preoperatively in 28 out of 32 cats. The use of antibiotic treatment could not be determined in 4 cats from the available medical records. The median hospitalization time following surgery was 6 days (range 3–11 days).

A positive urine culture was recorded from the intraoperative pyelocentesis sample in 4 out of the 32 cats (12.5%); *Escherichia coli* (*n* = 2), *Acinetobacter baumannii* (*n* = 1) and *Staphyloccocus spp* (*n* = 1) were isolated. The appropriate antibiotic treatment was administered for 3 weeks after antimicrobial susceptibility testing results. Antibiotic treatment administered, duration of treatment and dosage for each species isolated are provided in Table 1. Infections were treated successfully in 2 (both *E coli)* out of the 4 cats according to the results from an antimicrobial susceptibility test, performed on a urine sample from the SUB port 1 week after the antimicrobial treatment was stopped. Three of these 4 cats had undergone bilateral SUB device placement. Two of these 4 cats appeared to have persistent UTI with the same species isolated every 6 months during the follow-up period.

**Table 1:** Antibiotics administered, duration of treatment and dosage for each species isolated from the intraoperative pyelocentesis sample (4 cats). When used, the administration of marbofloxacin was preceded by another antibiotic treatment (amoxicillin clavulanic acid) and an antimicrobial susceptibility test

Cat	Species isolated	Antibiotics	Dose/route	Duration	Bacteriuria
1	*A. Baumannii*	Marbofloxacin	4 mg/kg PO SID	3 weeks - 4 more weeks	Persistent
2	*Staphyloccocus*	Amoxicillin clavulanic acid	15 mg/kg PO BID	3 weeks - 8 more weeks	Persistent
3	*E coli*	Amoxicillin clavulanic acid	15 mg/kg PO BID	3 weeks	Resolved
4	*E coli*	Amoxicillin clavulanic acid	15 mg/kg PO BID	3 weeks	Resolved

**Fig. 1:**
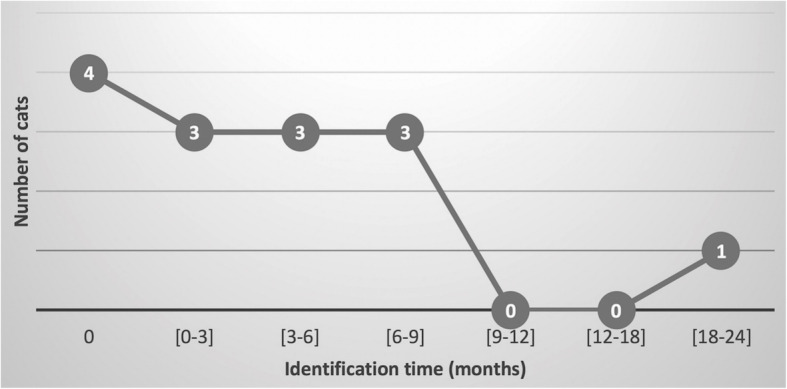
Number of cats with identification of a new positive urine culture over a two-year follow-up period (10/28 cats)

The median follow-up time after SUB device placement was 362 days (range 185–1312 days). Ten out of the 28 cats that did not have a positive culture at the time of surgery (35.7%) presented a positive urine culture during the follow-up period. Of those 10 cats, 9 (90%) had an initial positive urine culture identified during the first year of follow-up and 1 cat had a positive urine culture noted 582 days post-operatively. Positive urine culture kinetics graphs is provided in Fig. 1. Nine of them showed lower urinary signs (pollakiuria and hematuria) and one cat was asymptomatic but urinalysis identified bacteria during urine microscopic sediment evaluation. No pyelonephritis signs were suspected clinically or during the ultrasound examination. Median time from discharge to identification of the first positive urine culture was 159 days (range 8–703 days).

Of those 10 cats, 6 had a bilateral SUB device (4 with a 3-way port, 2 with 2 shunting ports) and 4 had a unilateral SUB device. Bacteriuria resolved with appropriate antimicrobial treatment in 6 out of 10 cats (60%) according to the results from an antimicrobial susceptibility test, performed on a urine sample from the SUB port 1 week after the antimicrobial treatment was stopped. Two out of these 6 cats had another episode of UTI (pollakiuria and hematuria) with identification of the same species (218 days and 249 days after the first episode, respectively). Bacteriuria resolved with antimicrobial treatment based on sensitivity. Organisms isolated (intraoperative and postoperative positive urine culture) are listed in Table 2.

**Table 2:** Organisms isolated during follow-up period

Bacteria	Number
*Escherichia coli*	1/10
*Enterococcus faecalis*	4/10
*Pseudomonas aeruginosa*	2/10
*Streptococcus canis*	2/10
*Enteroccoccus faecalis + Escherichia Coli*	1/10

Of the 4 cats whose bacteriuria did not resolve with appropriate antibiotic treatment, 3 had persistent UTI. One cat had subclinical bacteriuria (*Enteroccoccus faecalis and E coli*), so antibiotic treatment was stopped after three periods of treatment. The device was flushed every 6 months, no systemic clinical signs were noted and biochemical values were stable, urine culture was declined by the owners. However, this same cat developed a left subcapsular perirenal abscess next to the dacron cuff combined with a severe pyelonephritis 3.5 years following his initial surgery. A left nephrectomy was performed at that time. The cat died from a septic shock during the surgical procedure. One cat was euthanized because of the progression of the chronic kidney disease 448 days post-operatively. All remaining cats were alive at the time of the study.

There was a significant difference between urine culture negative and urine culture positive group in median hospitalization duration (5 days versus 6 days respectively, *P* = 0.022) and median body condition score (5/9 versus 4/9 respectively, *P* = 0.03). Cats with a longer hospital stay and with a lower BCS were more likely to have a positive urine culture during the follow-up period. There was no significant difference between urine culture negative and urine culture positive groups for the remaining variables evaluated. No significant difference was identified between the two groups for the progression of the chronic kidney disease (*P* = 0.25). No other associations could be found statistically in the criteria evaluated.

Clinical summary data are provided in Table 3.

**Table 3:** Urine culture results during follow-up compared with signalment, surgical and anesthesia duration and hospitalization duration

Culture	N	Variable	Mean	SD	Median	Minimum	Maximum
Negative (follow-up)	18	Age (years)	6.09	3.14	6.04	1.67	13.1
		Body condition score (/9)†	5.38	0.92	5	3	7
		Surgical duration (minutes)	106	26.64	105	50	150
		Anesthesia duration (minutes)	162	20.15	160	110	190
		Hospitalization duration (days)*	5	1.28	5	3	7
Positive (follow-up)	10	Age (years)	5.11	2.48	3.6	2.5	8.9
		Body condition score(/9)†	3.77	1,3	4	2	5
		Surgical duration (minutes)	97	19.64	100	72	135
		Anesthesia duration (minutes)	154.78	29.89	155	121	215
		Hospitalization duration (days)*	6.22	1.56	6	3	9

* There was a significant difference between negative and positive groups for median hospitalization duration in days (*P* = 0.022)

† There was a significant difference between negative and positive groups for median BCS (/9) (*P* = 0.03)

Overall, chronic bacteriuria was documented for 6 of 32 (18.8%) cats and 5 of 32 (15.6%) cats displayed clinicals signs suggestive of chronic UTI. Cats with an intraoperative positive urine culture were not more likely to have a positive urine culture during the follow-up period (*P* = 0.081).

Three cats died during the follow-up period, at 262, 448 and 1231 days. Two cats were euthanized following a deterioration in general condition due to persistent ITU, and one cat died during peri-renal abscess drainage surgery.

## Discussion

This is the first report attempting to evaluate the risk factors of positive intraoperative and postoperative urine culture in cats after SUB device placement for the treatment of feline ureteral obstructions. The present study identified 35.7% of new positive urine culture during the follow-up period. Overall, 15.6% of chronic UTI were identified in this cat population. Cats with a decreased BCS and a longer hospitalization duration had significantly increased risk for the development of positive urine culture after hospital discharge. However, positive intraoperative urine cultures were not related to the development of signs consistent with UTI during the follow-up period, and duration of surgery and anesthesia were not associated with positive urine bacterial cultures.

The use of a SUB device was recently reported in a large series of 134 cats with good outcomes [1]. Three studies reported that cats treated with SUB device had a 88 to 94% survival to discharge rate [1, 9] and a 1 year survival of 83% [7]. In the present study, survival to discharge rate was 100%. Three cats died during the follow-up period, at 262, 448 and 1231 days.

In previous studies, postoperative positive urine culture, obtained from the SUB port, was reported from 21 to 25% of the cases [1, 6, 7]. Our findings were slightly higher compared to those reported as we identified 35.7% of positive bacterial urine culture during follow-ups. All of the urine samples were obtained from the SUB port in order to avoid contamination. Bacteriuria resolved in 60% of the cases with appropriate antibiotic treatment which is consistent with the previously reported data of 50 to 78% of cats [1, 2].

As recommended by Kopecny et al. [6] a distinction between subclinical bacteriuria and UTI was made to determine the clinical relevance of infection and the necessity to administer antibiotics. This distinction allowed us to identify chronic UTI in 15.6% of cats which is much higher than the 8% previously reported in a study of 132 cats [1]. In our study, 3.1% of cats had subclinical bacteriuria, which is consistent with the 4.9% previously reported [1] but lower than the 12.5% reported in another study [6].

The effectiveness of extending post-operative antibiotic treatment could not be determined within the limits of this study. It appears in a study that post-operative antibiotic treatment administration decreased the risk for development of positive urine culture after discharge, with a median duration of 5 days of treatment [6]. In the present study all cats received prophylactic antibiotic treatment for 7 days until reception of bacterial culture results. The antibiotic treatment was stopped if the urine culture was negative and pursued for 3 weeks if positive, with the antibiotic based on sensitivity [10, 11]. Antibiotic use following surgery may be associated with a decrease of bacterial colonization of the SUB device [6].

The most commonly isolated pathogens were *E coli*, *E faecalis* and *P aeruginosa*. This is consistent with other studies reporting SUB placement but also with cats with bacteriuria diagnosed by urine culture where *E coli and Enterococcus species* are the most common feline urine pathogen cultured [1, 6, 12–15]. *Enterococcus* species was significantly more common in cats with subclinical bacteriuria [12, 15], this was however not the case in another study with ureteral devices [6]. These characteristics could lead to an absence of antimicrobial treatment depending on the clinician. However, *Enterococcus* spp. has an ability to form biofilms [13] and implant surfaces act as substrates for bacterial biofilm formation [16], which will be recalcitrant to antibiotic therapy. *E faecalis* was isolated in 2 urine culture in our study and both cats were treated with appropriate antibiotic treatment which led to a resolution of their clinical signs. A left subcapsular perirenal abscess combined with a severe pyelonephritis was diagnosed 3.5 years following initial surgery in a cat with subclinical bacteriuria (*E faecalis and E coli*). The ability of *Enterococcus* spp. to form biofilms associated with a decrease of host immune response could have led to the development of the abscess. However, the bacterial strain responsible for the perirenal abscess was not identified as no bacterial culture was performed since the cat died during the nephrectomy. Although causative bacteria is unknown, this long-term complication has never been described before and emphasizes the interrogation to treat subclinical bacteriuria.

Berent et al. (2018) found that cats with a positive urine bacterial culture result or bacteriuria during surgery of SUB device placement were significantly more likely to have bacteriuria at some time after surgery and to have persistent UTI. In the present study, these findings were not observed. However, our results come from a small number of cats and should therefore be interpreted with caution.

Of the 32 cats included, the SUB device type was compared with positive bacterial urine culture and no significant predilection were found. However, this absence of significant difference between the groups in our study may reflect a type II error. The 3-way port allows to connect both kidneys to a single bladder catheter for bilateral ureteral obstructions. It is ideal for bilateral ureteral obstructions in compromised patients because it reduces the anesthesia duration [8]. Risk factors for infection and mineralization of this type of device have not been evaluated and compared to single port device, but there are theoretically more potential risks from using this device as a mineralization or a kink of the bladder catheter negatively impact both kidneys. More recently, new port design with a larger chamber of silicone were marketed. This design is supposed to create more turbulence during flushing procedure. Further studies evaluating the 3-way port and this new chamber design are warranted. Additionally, the therapeutic use of tetrasodium ethylenediaminetetraacetic acid solution (tEDTA) has been evaluated during SUB flushing to prevent and treat biofilm infection and to prevent and treat mineralization occlusion [1]. tEDTA is effective at eradicating biofilms formed by microorganisms from central venous catheters in human patients [1, 17]. In a recent study, the implementation of routine irrigation of the SUB device using 2% tEDTA led to a decrease in both infection and mineralization rate associated with SUB devices. In 8 SUB device occlusions secondary to mineralization, obstruction resolved after treatment with tEDTA infusion [18].

There was a significant difference in hospitalization duration between positive and negative bacterial urine culture group (5 days versus 6 days, *P* = 0.022). The use of indwelling urethral catheter after SUB device placement to quantify urine output is associated with positive postoperative urine bacterial culture results in 2 studies [1, 14]. However, in our institution, use of non-absorbent cat litter improve the ability to accurately quantify urine output, which explain that no cats in this study had an indwelling urethral catheter.

Evaluation of health-care associated infection, which are infections animals get while they receive health-care for another condition, in critical care units of small animal referral hospitals found that 12% of cats have had one or more nosocomial infection occur during hospitalization [19]. One of the risk factors found to have a positive association with development of a nosocomial infection was longer hospital stay [19]. However, median time from discharge to identification of the first positive urine culture was 159 days (range 8–703) in this study. If the bacteriuria was caused by a contamination during hospitalization, it should have been diagnosed in the first few days after discharge. Pathogen isolated were mostly *E coli* and *E faecalis*. These organisms are known to form bacterial biofilms and to grow more slowly than planktonic state bacteria [20, 21]. While 68% of ureteral stent in human patients become colonized in one study, only 27% of bacteriuria was documented [22]. Therefore, a negative urine culture does not rule the possibility of SUB device colonization. Interestingly, if antimicrobial susceptibility testing is based on bacterial culture derived from planktonic bacteria, antimicrobial agent might be ineffective against bacteria in biofilm which differ in behaviour and in phenotypic form from planktonic bacteria [21].

There was also a significant difference in body condition score between urine culture-negative and urine culture-positive groups (5/9 versus 4/9, *P* = 0.03) which is consistent with the risk factors evaluation for development of UTI in cats [14]. A risk factor that was also identified with increased odds of UTI was a decreased BCS. It may be explained by the advanced status of concurrent conditions predisposing to UTI via patient debilitation, weakened immune system and increased susceptibility to infection [14].

No significant differences were identified between urine culture-negative and urine culture-positive groups concerning the progression of chronic kidney disease. This is consistent with a previous study in cats with hyperthyroidism, diabetes mellitus and chronic kidney disease which reported no association between UTI and increased serum creatinine concentrations [23]. In another study, the presence of clinically relevant chronic kidney disease (IRIS stage 2–3) was not identified as a risk factor for the development of a positive urine culture and UTI occurred independently of concurrent chronic kidney disease [6].

This study has a number of limitations, related to its retrospective nature. A small number of cats met the inclusion criteria. The small group sizes may have limited the power, and hence differences in studied parameters, such as the surgery or anesthesia duration, SUB device type and the progression of the chronic kidney disease, may have reached statistical significance in a larger population. Therefore, this absence of significant difference between the groups in our study may reflect a type II error. The duration of antimicrobial treatment was standardized between the animals in this study, but remains dependent on the wishes of each institution. Selection bias is possible because urine culture may have been performed in cats with clinical signs compatible with UTI. Asymptomatic bacteriuria might have been missed with only a urinalysis. Further studies evaluating the effect of post-operative antimicrobial administration in a larger group of cats with SUB device placement are necessary.

## Conclusions

Following SUB device placement surgery, positive urine cultures were documented during follow-up period in one third of the cats. Bacteriuria resolved with appropriate antimicrobial treatment in more than half of the cases. Chronic UTI were documented in one fifth of the cats and subclinical bacteriuria in one case. Risk factors identified for positive urine culture following surgery were hospitalization duration and a decreased body condition score. However, duration of surgery and anesthesia were not risk factors. Similarly, positive intraoperative urine cultures were not associated with the development of signs consistent with UTI during the follow-up period. Further studies evaluating the use of post-operative antimicrobial treatments and therapeutic use of tEDTA solution to prevent biofilm formation in cats with ureteral devices are needed.

## Data Availability

The dataset used and/or analysed during the current study are available from the corresponding author on reasonable request.

## Abbreviations

SUB: Subcutaneous ureteral bypass
tEDTA: Tetrasodium ethylenediaminetetraacetic acid solution
BCS: Body condition score
IRIS: International Renal Interest Society staging
ISCAID: International Society for Companion Animal Infectious Diseases
